# Does Choose & Book fail to deliver the expected choice to patients? A survey of patients' experience of outpatient appointment booking

**DOI:** 10.1186/1472-6947-8-36

**Published:** 2008-08-01

**Authors:** Judith Green, Zoe McDowall, Henry WW Potts

**Affiliations:** 1 The Hillingdon Hospital, Pield Heath Road, Uxbridge, UB8 3NN, UK; 2 CHIME, University College London, Whittington Campus, Highgate Hill, London, N19 5LW, UK

## Abstract

**Background:**

Choose and Book is a central part of the UK Government patient choice agenda that seeks to provide patients with a choice over the time, date and place of their first outpatient appointment. This is done through the use of a computerised booking system. After a 2004 pilot study, Choose and Book was formally launched in January 2006. This is the first study of patient experience of Choose and Book since then.

**Methods:**

A questionnaire survey of reported experience of choice over the time, data and place of appointment, carried out in a National Health Service hospital in London. 104 patients at their first outpatient appointment completed the questionnaire, consisting of a consecutive series of patients referred through Choose and Book and a sample referred through the conventional booking system.

**Results:**

Among the Choose and Book patients, 66% (31/47; 95% CI 52 to 78%) reported not being given a choice of appointment date, 66% (31/47; 95% CI 52 to 78%) reported not being given a choice of appointment time, 86% (37/43; 95% CI 74 to 94%) reported being given a choice of fewer than four hospitals in total and 32% (15/47; 95% CI 20 to 46%) reported not being given any choice of hospital.

**Conclusion:**

In this study, patients did not experience the degree of choice that Choose and Book was designed to deliver.

## Background

Choose and Book is the National Health Service's electronic booking system for first outpatient appointments in secondary care [1]. It is a key project within NHS Connecting for Health and central to the UK Government's patient choice agenda [2]. By phoning an appointments line, booking over the Internet, or booking at the GP surgery, patients have a choice of time, date and place for their appointment. Choose and Book is an international exemplar of both the introduction of a large-scale medical informatics system and of a government policy to deliver patient choice.

Choose and Book enables *Choice at referral *[3], which was the first commitment to providing patients with more choice about when and where they receive treatment [4]. Since January 2006, barring certain exceptions, all patients requiring elective treatment should be offered the choice of at least four providers, once their GP has decided a referral is required [1]. Choose and Book also furnishes a choice of appointment date and time, a central element of the business case for electronic booking [5]. Since April 2008, the scheme has been further expanded with the Free Choice policy under which most patients should be able to choose from any secondary care provider (NHS or independent sector) across England.

In Choose and Book, the referring clinician creates an Appointment Request by selecting a shortlist suited to the patient's clinical needs and preferences from a Directory of Services. Patients then book from a list of available slots in one of three ways:

i. Patient calls the Booking Management Service quoting a unique booking reference number (UBRN) given to them by the GP;

ii. Patient accesses Choose and Book through the Web-based Patient Portal within NHS Healthspace [6] and books for themselves, using their UBRN; or

iii. As part of or following the consultation, the GP or one of the practice staff uses the Choose and Book system to make an appointment while the patient is in the surgery.

Patient choice has been heralded as the driver for transforming the NHS and a means of "meeting patient expectations" ([7], p. 56). In combination with the new NHS funding mechanism of Payment by Results, where money follows the patient, patient choice is reasoned to provide hospitals with an incentive to improve the quality of their services to attract patients. It is cited as the solution to much that is presently wrong with the NHS from excessive waiting times to even car parking issues [8].

Current theories around choice empowering patients and driving up standards is, for many, convincing. The notion that the concept of consumerism can be applied to healthcare seems plausible given patient's desire for choice [9-12]. However there are alternative views at both the macro level and at the micro level of patient experience [13-16]. 'Choice overload' may lead to bewilderment and anxiety, particularly for patients without access to, or skill to understand, information to make decisions about choices on offer [13,14]. There are questions whether the choices proposed are those that patients desire [17,18] and even over demand for choice itself. Patients' attitudes to choice are inconsistent and variable, depending on their individual circumstances, the types of choice and when they are offered [19]. For example, 89% of survey respondents agreed that access to a good local hospital was more appropriate than having more hospitals from which to choose [20].

The case for a choice of appointment date and time has been less contentious and is expected to produce a reduction in non-attendance ([5], p. 93), as well as satisfying patient demands.

Where choice has been introduced in pilot schemes, it has proved popular with patients. Pilots of the earlier *Choice at six months *scheme [21] demonstrated high take up rates [22,23] with a large majority of participants stating that they would recommend the scheme [24], although these may not be representative of *Choice at referral*. A pilot scheme for *Choice at referral *was successfully run [25]. Although there is some consensus among these different studies, they highlight that patients' attitudes to choice are variable and depend on the types of choice and when they are offered. The national inception of Choose and Book in 2006 presented an opportune time to assess patients' experiences.

To 3 April 2006, Choose and Book had been used for 261,983 bookings, 12% of the total [26]. It is estimated that bookings are growing by at least 40% *per *month. The take up of the system is estimated to be a year behind schedule, due in part to the extension of the scope of the originally designed e-booking system to support *Choice at referral*. There has been considerable bad feeling associated with Choose and Book with criticism about risks to patient confidentiality, reliability and speed [27-32].

## Methods

Using a structured questionnaire in two variants, we evaluated attitudes and experiences among patients referred to the Hillingdon Hospital (Hillingdon site only) through Choose and Book or through the conventional booking process, Partial Booking. The Hillingdon Hospital NHS Trust is based on two sites (Hillingdon and Mount Vernon) in outer London, serving a population of over 300,000. Hillingdon Hospital is the only acute hospital in the London borough of Hillingdon.

Partial Booking is where a GP sends a written letter of referral to a specified hospital. The choice of the hospital is made by the GP with as much consultation with the patient as the GP chooses. The hospital then writes to the patient to acknowledge their referral and advises them of the anticipated waiting time. This letter requests that the patient phone the hospital booking centre to arrange their appointment.

We did not find any existing validated measures that could be used here. Patient questionnaires were developed based on a review of the literature and, in particular, consideration of the quantitative research methodology used in Patient Choice pilot studies [23,33]. Initial drafts were developed and reviewed by the research team and operational staff (Outpatient Booking Co-ordinators) at the Hillingdon Hospital. Amendments were made and a second draft version of each questionnaire then piloted among a small number of initial respondents within the study, following which some minor adjustments were made. Full questionnnaires are given in the Appendix; additional data to those reported here were also collected.

Using the Trust's Patient Administration System (PAS), a consecutive series of new patients referred through Choose and Book was identified over a planned three-month period. Three months was chosen as a compromise between providing a useful sample size and limited researcher resource. Patients were approached in clinics while waiting to be seen. This was done in preference to a postal survey so as to minimise response bias, where we were concerned patients with particularly negative experiences would be more likely to respond, and to better be able to include patients with poorer English literacy. While waiting at the hospital is also a salient time for patients to recall their booking experience. A matched sampling methodology was adopted recruiting patients who were present in the same outpatient waiting area at the same time and who had come through Partial Booking. However, for logistical reasons, it was not always possible to interview a matched patient. To increase the Partial Booking sample size, during the same period, interviewers attended clinics on additional days and interviewed all new patients in attendance.

Patients were recruited by JG, ZM or two assistants. They were given a copy of a patient information sheet, which gave them the option not to proceed. Consenting subjects were asked to complete one of two questionnaires while waiting in clinic for their appointment, the questionnaire being dependent on their referral type. Participants were assisted with the questionnaires as necessary, with regular meetings between the data collectors to ensure that any such assistance was consistent and unbiasing. Prior arranged translation arrangements for the consultation were used to facilitate completing the questionnaire; these included a British Sign Language signer and interpretation through family members/escorts.

The study was approved by the Hillingdon Hospital NHS Trust Research and Development department at the time as part of an audit. In seeking to publish, we sought advice from the NHS Local Research Ethics Committee, whose Chair confirmed that the study is not considered to be research according to the National Research Ethics Service's guidelines and thus had not needed Research Ethics Committee approval.

## Results

### Description of the sample

**Figure 1:**
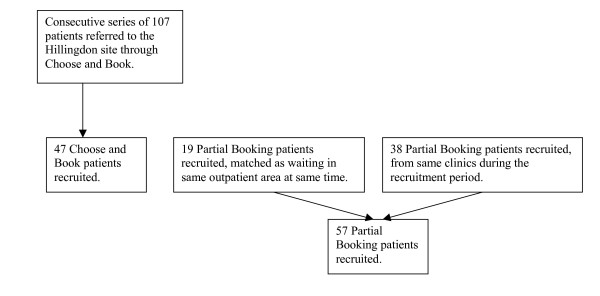
Flowchart showing study recruitment.

A total of 104 patients took part in the study between 4 May and 9 August 2006. Of these, 47 were Choose and Book patients. This represents 44% of the 107 total Choose and Book patients seen at the Hillingdon site between these dates. A further 57 patients were referred through the conventional Partial Booking referral process, 19 matched and 38 from additional clinics. Figure 1 presents a flowchart of recruitment.

Data were collected on participants' gender, age, ethnic group and specialty division; see Table 1. Age data was Normally distributed; other variables are categorical. The two groups were not statistically significantly different on gender, age or ethnic group. The sample was compared with data obtained on all first outpatient attenders at the Trust in the same time period: χ^2^-tests showed no statistically significant difference by gender, age category or ethnic group.

**Table 1:** Background variables

Variable	Categories	**Choose & Book **(*n *= 47)	**Partial Booking **(*n *= 57)	TOTAL	Test comparing groups (excluding missing data)
Gender	Female	29	38	67	Fisher's exact *p *= 0.7
	Male	18	19	37	
Age	16–29	10	9	19	Mann-Whitney *z *= 1.5, *p *= 0.1
	30–44	6	18	24	
	45–59	11	17	28	
	60–79	16	11	27	
	80+	4	2	6	
Ethnic group	White British	30	41	71	Fisher's exact *p *= 0.6
	South Asian	9	8	17	
	Other	3	7	10	
	*missing*	5	1	6	
Specialty division	Ambulatory Care	10	29	39	Fisher's exact *p *= 0.008
	Surgery	14	8	22	
	Women & Children	8	10	18	
	Medicine	11	6	17	
	*missing*	4	4	8	
"How important to you is being given choice over where you go to receive hospital treatment?"	Very important	21	27	48	Mann-Whitney *z *= 0.8, *p *= 0.4
	Important	12	17	29	
	Slightly important	3	4	7	
	Not important at all	0	4	4	
	*missing*	11	5	16	

There was a significant difference between Choose and Book and Partial Booking patients in terms of the specialty division they were under, with more of the Partial Booking patients under Ambulatory Care (Table 1). Participants were also asked how important it was to them to be offered a choice of hospital and the two groups showed no statistically significant difference (Table 1).

At the beginning of the questionnaire, patients were asked whether they had been aware, before their GP appointment, of their entitlement to choose to which hospital to be referred. Overall, 63% (65/104; 95% CI 53 to 71%) said they had not; there was no statistically significant association between prior knowledge and referral method (Fisher's exact *p *= 0.3).

### Experience of choice

Patients were asked if they felt they had been given any choice over their outpatient appointment. No guidance was given about what choice meant. A series of questions then probed the nature of choice respondents felt had been offered. Overall, 52% (53/102; 95% CI: 42 to 61%) of patients felt they had been given choice over their appointment: Choose and Book patients more often than Partial Booking patients. Choose and Book patients also reported being given a choice of hospital more often and being offered a greater number of hospitals than Partial Booking patients. However, there were no statistically significant differences between the two groups on being given a choice of appointment date or time (Table 2).

**Table 2:** Perception of choice by referral method

Question		Categories	**Choose & Book ** (*n* = 47)	**Partial Booking ** (*n* = 57)	TOTAL	Test comparing groups (excluding missing data)
"Do you consider that you were given any choice over your appointment?"		Yes	30	23	53	Fisher's exact *p *= 0.015 (*no *and *unsure *combined)
		No	13	31	44	
		Unsure	2	3	5	
		*missing*	2	0	2	

"Were you given a choice of the following?"	Hospital	Yes	32	11	43	Fisher's exact *p *< 0.001
		No	15	46	61	
	Appointment Date	Yes	16	17	33	Fisher's exact *p *= 0.7
		No	31	40	71	
	Time of Appointment	Yes	16	14	30	Fisher's exact *p *= 0.4
		No	31	43	74	

Number of hospitals offered		1 – no choice	15	47	62	Mann-Whitney *z *= 5.3, *p *< 0.001
		2	16	7	23	
		3	6	1	7	
		4	6	0	6	
		Can't remember	4	0	4	
		*missing*	0	2	2	

Focusing just on the Choose and Book patients, 29% (13/45; 95% CI 17 to 43%) reported not being given any choice, 32% (15/47; 95% CI 20 to 46%) reported not being given a choice of hospital, 66% (31/47; 95% CI 52 to 78%) reported not being given a choice of appointment date, 66% (31/47; 95% CI 52 to 78%) reported not being given a choice of appointment time, and 86% (37/43; 95% CI 74 to 94%) reported being given a choice of fewer than four hospitals in total. In all, only one Choose & Book patient (2%; 95% CI 0 to 10%) stated that they had been offered a choice of four hospitals with a choice of appointment date and time.

Choose and Book patients were asked how their appointment had been booked. Just over half (53%, 25/47) were booked within the GP surgery, with 83% of these (20/24) booked by the GP versus the remainder by non-clinical staff (one response missing). Another third (36%, 17/47) were booked by the patient calling the NHS Direct Appointment Booking Line. Booking on-line accounted for 11% (5/47). How appointments were booked did not statistically significantly vary by gender, age or specialty division. However, there was a relationship with ethnic group (Fisher exact *p *= 0.007) with all the south Asian patients booking within the GP surgery.

There was a statistically significant association between how Choose and Book patients booked their appointment and whether they reported being given a choice of appointment date and time. Those booking within the GP surgery were least likely to report having a choice and those online, most likely (Table 3).

**Table 3:** Perception of choice by Choose and Book booking method

Question		Categories	**Within GP surgery**	**Call centre**	**Online**	TOTAL	Fisher exact test comparing groups
"Do you consider that you were given any choice over your appointment?"		Yes	13	14	3	30	*p *= 0.07 (*no *and *unsure *combined)
		No	11	2	0	13	
		Unsure	1	1	0	2	
		*missing*	0	0	2	2	

"Were you given a choice of the following?"	Hospital	Yes	17	12	3	32	*p *= 1.0
		No	8	5	2	15	
	Appointment Date	Yes	5	6	5	16	*p *= 0.003
		No	20	11	0	31	
	Time of Appointment	Yes	21	9	1	16	*p *= 0.008
		No	4	8	4	31	

Those participants who indicated that they had been given a choice over their appointment were asked how satisfied they were with the experience (Table 4). Overall, 14% (6/44; 95% CI 6 to 26%) of patients were dissatisfied with the experience of booking their appointment. Among those who reported being given a choice, there was no statistically significant difference between Choose and Book and Partial Booking patients. For both groups, the median response was "fairly satisfied" (95% bootstrapped CI [34]: "very satisfied" to "fairly satisfied").

**Table 4:** Satisfaction with choice

Question	Categories	**Choose & Book ** (*n* = 30)	**Partial Booking ** (*n* = 23)	TOTAL	Mann-Whitney test comparing groups
For those who reported being given a choice: "Overall, how satisfied or dissatisfied were you with the experience of choosing your hospital?"	Very satisfied	11	9	20	*z *= 0.2, *p *= 0.9
	Fairly satisfied	9	9	18	
	Fairly dissatisfied	3	1	4	
	Very dissatisfied	1	1	2	
	*missing*	6	3	9	

## Discussion

A sizable proportion of patients in this study referred through Choose and Book do not consider that any choice was available to them. That over a quarter (29%) of Choose and Book patients felt they had not been offered choice is in itself striking, but that even higher proportions did not perceive that they had been given a choice of hospital (32%), appointment date (66%) or appointment time (66%) means that, even when patients are offered choice, it does not match Government intentions. While the confidence intervals for these results are wide, even their lower limits are surprisingly high. The clearest demonstration of this misalignment is that, of the complete study sample, only one patient stated that they had been offered a choice of four hospitals with a choice of appointment dates and times, that which Choose and Book supposedly offers to everyone.

As far as we know, this is the first study of patients experiencing the live implementation of Choose and Book. This is a small study; non-significant statistical test results in comparisons between the Choose and Book and Partial Booking patients should not be over-interpreted given the limited power. It must be recognised that, at the time of the study, not all outpatient clinics at Hillingdon Hospital were Choose and Book enabled and a limited number of GPs were using Choose and Book locally, so the Choose and Book patients surveyed are not necessarily representative of the population coming through the system over the next few years. Restricted interviewer resource prevented achieving a complete consecutive series of Choose and Book patients. Matching between Choose and Book patients and Partial Booking patients was also limited. However, the sample group was representative of the total first outpatient population of Hillingdon Hospital NHS Trust in terms of gender, age and ethnicity.

The methodology was retrospective in nature and relies on patients' recall. For a true account of how choice is being enacted by healthcare providers and received by patients, observational studies are required of patient/GP consultations.

It might be anticipated that people with current health issues would have greater awareness of NHS policy than the general public. We found 63% (95% CI 53 to 71%) of patients had no prior knowledge of patient choice compared with a poll showing 80% of British residents aged over 40 knew little or nothing about choice reforms [33].

Our results suggest the Choose and Book booking process may be one factor contributing to the discrepancy in patients' experience of choice. The booking method influences the degree of choice patients perceived. Those booking appointments at the GP surgery are less likely to consider that they have been given a choice over the date or time of their appointment than patients booking their own appointments through the call centre or over the Internet. This may be due to the manner in which the options are either expressed by staff or understood by the patient. For example, where options are framed by the GP as a package of a hospital, a date and a time, any focus, by the patient or the GP, on one individual component may overshadow that further choice has been offered. The reality of there being low awareness of choice among the general public could also mean that patients tend to accept the first date/time offered and primary care staff, therefore, do not present further choices. In contrast, Internet booking involves discrete stages for each of the three choice elements.

Comments made by participants during data collection suggest, however, that booking online is not always successful. A number of respondents spoke of initially trying to book their appointment over the Internet, but technical problems forced them to call the booking line or go back to their GP to book their appointment. This could partly explain why the proportion of patients booking online is small and suggests that, for Choose and Book to deliver the intended scope of choice, online booking needs to be improved.

Patients' perception of the scope of choice offered may also be influenced by their own priorities. Comments made by a number of participants suggest that being offered an alternative hospital with, for example, an unsatisfactory waiting time did not consitute a real choice for them. The same was suggested of hospitals with long travel times. For other patients, the fact that their preferred hospital was not on the menu of providers compromised their perception of being offered choice. Ancedotal reports suggest that the timing of clinics is such that the choice of appointment time and day that patients want cannot be delivered, leading to disappointment.

Neither patients' nor GPs' behaviour necessarily conforms to models of rational choice economics. For example, they tend to show loyalty to their familiar healthcare providers even when they may not offer the best quality care [14]. Our results here may be an expression of that cultural barrier to choice. Given the asymmetric doctor/patient relationship, patients may also choose not to choose: even competent adults may prefer to delegate their choice of treatment to someone, typically a health professional, whom they regard as better informed to take the decision on their behalf [14,18,35]. The role of the GP is anticipated to remain fundamental to patient choice, which places potential constraints on how *Choice at referral *is implemented as the GP can decide how to frame options available to the extent that not all the options are perceived by the patient as being available.

## Conclusion

The findings from this preliminary study suggest that Choose and Book did not deliver choice as portrayed in UK government policy to this patient comunity. A key question for researchers now must be whether these findings generalise across the country. There is also a need for prospective methodologies looking at patient behaviour and experience of Choose and Book. If the findings do stand up to replication, there could be consequences for the programme: for example, if the majority of patients are not experiencing a choice over appointment time and date, will it produce the expected reduction in non-attendance? One could make recommendations that the availability of choice needs to be further promoted; and technical issues need to be addressed. However, as with other policies to increase patient choice, substantial investment may be required in restructuring healthcare services if Choose and Book is failing to deliver [35].

Choice is only meaningful if there are realistic options and an experience of choice. We suggest our results reveal both a symptom and a cause: the lack of experienced choice may be a symptom of a lack of meaningful choice in the system, while aspects of the system's design may cause patients to experience less choice than intended.

Our results paint a different picture to the case studies on the Choose and Book website [36]. While our findings about Choose and Book need replicating, they more generally match prior studies showing the public is not experiencing the intent of UK government policy on choice [17,37]. Consumerist models of choice driving quality improvements fail if patients are not exercising that choice. Understanding the discordance between experience and policy intent is crucial to the success of the patient choice agenda. We suggest that consideration needs to be given as to whether choice of hospital should be the focus of patient choice and whether the nature of NHS services, or healthcare services in general, are such that a meaningful choice of place, date and time can ever be delivered.

## Competing interests

The authors declare that they have no competing interests.

## Authors' contributions

JG conceived the original study, which was developed with HP. Data collection was by JG and ZM. The paper was drafted by HP and JG and all three authors were involved in critically revising the final version.

## Pre-publication history

The pre-publication history for this paper can be accessed here:


